# Oral health status of nursing staff in Ilembula, Wanging’ombe District, Njombe region, Tanzania: a cross-sectional study

**DOI:** 10.1186/s12903-022-02064-x

**Published:** 2022-05-09

**Authors:** Tobias Bensel, Imke Erhart, Simon Megiroo, Werner Kronenberg, Wolfgang Bömicke, Sebastian Hinz

**Affiliations:** 1 Department of Prosthodontics, University of Halle, Magdeburger Straße 16, 06112 Halle (Saale), Germany; 2 Health Department, ELCT/NORTH CENTRAL DIOCESE, P. O. Box 16173, Arusha, United Republic of Tanzania; 3 Ilembula Lutheran Hospital, P.O. Box 14, Ilembula, United Republic of Tanzania; 4 Department of Prosthetic Dentistry, Heidelberg University Hospital, Im Neuenheimer Feld 400, 69120 Heidelberg, Germany

**Keywords:** Caries incidence, Tanzania, General oral health, Epidemiology, Nursing staff

## Abstract

**Background:**

Owing to the reduced dental treatment infrastructure in the Tanzanian highlands, maintaining good oral health is a challenge for not only the general population but also individual professional groups. In this study, the caries prevalence and, subsequently, the prosthetic treatment needs of the nurses of the Ilembula Lutheran Hospital (ILH) and Ilembula Institute of Health and Allied Sciences (IIHAS), Tanzania, were investigated.

**Materials and methods:**

One hundred and sixty-eight ILH and IIHAS nurses and nursing students (87 women, 81 men; age 23.1 ± 6.1 years, range 18–58 years) participated in this cross-sectional study conducted in February 2020. The participants were examined at the dental office of ILH. The Decayed, Missing, and Filled Teeth (DMF/T) Index, Simplified Oral Hygiene Index, and details regarding edentulism, nutrition habits, and socioeconomic factors were collected. Linear regression and binary logistic regression were used for statistical analysis.

**Results:**

The mean DMF/T-Index was 6.30 ± 4.52. In 7.14% of the investigated nurses, no dental plaque was detected. An enhanced prosthodontic treatment (Kennedy Class III) demand was identified in 31.50% of the participants, and 4.80% of the participants required treatment for acute malocclusion. Oral hygiene products were used by 99.4% of the patients.

**Conclusions:**

The current oral health situation of the study participants showed a moderate restorative and prosthetic treatment demand in the rural area of Tanzania. The development of an interdisciplinary oral health prophylaxis system could be a means to remedy this situation.

## Background

The most common global diseases associated with social and behavioral factors are dental caries and periodontal disease [1]. Compared to Western industrialized countries, the prevalence and severity of dental caries in Tanzania are still lower [2]. Poor oral health, poverty, systemic diseases, and inequality are the contributing factors to periodontal diseases, as recognized by the World Health Organization (WHO) [2]. Presently, approximately 3.5 billion people worldwide suffer from reduced oral health conditions, including caries, periodontal diseases, and edentulism [3].

Since the trade liberalization of East Africa, there has been an increase in the consumption of refined sugar foods [4]. This has led to an increase in the prevalence and severity of dental caries among Tanzanian communities [5]. In Tanzania, the dentist-patient ratio is approximately 1:360,000 [6]. However, there are additional differences in reported proportions owing to unevenly distributed accessibility to dental care between urban and rural regions [6]. This includes limited infrastructure and oral health care providers, who are mostly short-term trained, serving in the public sector, especially in rural areas. Therefore, access to high-quality healthcare services is limited in rural areas of low-income countries such as Tanzania [7]. However, the supply of high-sugar foods is increasing in the rural areas of Tanzania [5]. Given the limited dental care infrastructure, it is imperative to implement community-based caries prevention programs in rural areas [8]. Additionally, given the lack of dentists in these areas, nurses could play an important role in supporting caries prevention programs. Nurses stay in constant contact with their patients and are the main module of community health welfare at their respective operation sites. Therefore, nurses should be trained on basic oral health knowledge and authorized to support local dental services in rural areas for caries prevention. This should lead to a basic promotive oral health service to the rural community that the nurses are serving. To achieve this long-term goal, the first step would be to study the oral health status of nurses in rural Tanzania to evaluate the level of awareness of dental caries and oral health. Compared to Western industrialized countries, the incidence of dental caries is lower in Tanzania. This is also true for the apprentices of health professions in additional regions of Tanzania [5]. Currently, there are no studies available on the oral health status of nursing staff in rural areas of southwest Tanzania.

The aim of the current study is to assess oral hygiene, oral health status, orthodontic and prosthodontic treatment need in nursing staff of the ILH and IIHAS in order to determine if nurses in rural areas without adequate dental treatment facilities could generally screen and educate their patients regarding oral health-related diseases and how to prevent them.

## Materials and methods

### Participants

A total of 168 adult nurses and nursing students were eligible for participation and all of them (87 women, 81 men; age 23.1 ± 6.1 years, range 18–58 years; median age 22.0 years) participated in this cross-sectional study. They were a sample of the employees of the IIHAS and ILH, who were available for participating in the oral examinations during their working time and have reached the minimum age of 18 years. Pregnancy was not an exclusion criterion. Data were collected in February 2020. All the participants provided their informed consent before participation.

### Study site

Ilembula is situated in Wanging’ombe District in the Njombe region in the southern highlands of Tanzania. The IIAHS is located at the ILH, which is a Designated District Council Hospital. ILH is a health facility that belongs to the Evangelical Lutheran Church in Tanzania (ELCT)—Southern Diocese (SD) and has operated as a hospital since 1950. The ILH is one of four hospitals of the Iringa-region with a commuter area of up to 2.7 million inhabitants (Morogoro-Mbeya-Region). In 2017, a total of 35.544 patients were treated in the ILH (Ilembula Lutheran Hospital Annual Report 2017).

### Data collection

Data collection was based on standardized case report forms and consisted of a questionnaire and a clinical oral examination according to WHO guidelines. The questionnaire used was structured close-ended. The study supporting administrator was bilingual (Kiswahili and English) and was being held in readiness to clarify any occurring questions regarding to the questionnaire or any other component of the investigation straightforward in the native language of the study participants. The following data were collected using the questionnaire: (1) personal data: age, gender, and nursing specialization (if present); (2) medical and dental history: general diseases, infectious diseases, impairment of wound healing, medication intake, and existing pregnancy (for women); (3) oral homecare: frequency and utensils; (4) luxury foods: frequency and consumption of alcohol, tobacco, coffee, tea, sugar-sweetened beverages, and food; (5) frequency of daily meals.

### Oral health data collection

The study included a one-time examination of the oral cavity of the participants. Clinical examinations were carried out by trained and calibrated dentists (TB, IE) in a classroom with natural daylight and a headlight as sources of illumination and with an assistant recording the observations. According to the WHO recommendation for oral health examinations, instrumentation included the following components: dental mouth mirror, dental probe, WHO probe, rubber gloves, and mouthguard. All instruments used were disposable, and they were discarded after each participant’s examination. The following data were collected clinically: the numbers of decayed, missing, and filled teeth were documented according to the decayed, Missing, and Filled Teeth (DMF/T) Index. The WHO definition was used to detect caries. Oral hygiene was measured using the Simplified Oral Hygiene Index (OHI-S) to determine the presence of plaque (debris) and calculus. It was calculated for six tooth surfaces. The buccal surfaces were inspected in teeth 11, 16, 26, and 31, and lingual surfaces in teeth 36 and 46. The categories and corresponding scores of the OHI-S were as follows: good (0.0–1.2), moderate (1.3–3.0), and bad (3.1–6.0). To investigate the malocclusion of the participants, the basic Angle classification was used; that is, sagittal molar occlusion was detected. Additionally, the following intraoral characteristics were described if present: presence of a medial diastema, gingival recession, and dental attrition. The degree of attrition was assessed by the following criteria: No loss of enamel surface characteristics versus loss of enamel surface characteristics. Partial edentulism was described using Kennedy’s classification. The Kennedy classification is a topographic classification that evaluates each jaw separately. Edentulous arches are divided in four major classes (class I: bilateral shortened row of teeth, class II: unilateral shortened row of teeth, class III: interrupted dental arch by an interdental gap, class IV: interdental gap of the entire anterior region). The further subdivision of the major classes I-III is effected according to the number of existing gaps (subgroup 1: one additional interdental gap, subgroup 2: multiple interdental gaps, subgroup 3: multiple interdental gaps with limited residual teeth).

### Data analysis

Statistical analysis was performed using the Statistical Package for Social Science (SPSS, version 25.0; SPSS Inc., Chicago, IL, USA). Linear regression was used to analyze the factors associated with DMF/T and OHI-S. In addition, binary logistic regression analysis was performed. Differences were considered significant at a *p* value of ≤ 0.05. Due to the explorative nature of the study, *p* values were interpreted descriptively only.

## Results

### Questionnaire—general medical history and dental medical history

The results with regard to general medical history and dental medical history can be taken from Table 1. In the vast majority of study participants, the general medical history was unremarkable (n = 163, 97.0%). Own oral hygiene products were used by almost all (n = 167, 99.4%) participants. Intraoral symptoms during oral homecare were reported by a minority of participants (n = 48, 28.6%). Dental treatment measures included direct fillings, tooth extractions, and dental check-ups and were reported by nearly one-third (n = 49, 29.2%) of the participants that they had sought dental treatment within the two years prior to study participation.

**Table 1:** Distribution of nursing staff’s responses regarding their general medical history and dental medical history

	Gender						
	Women	Age in years		Men	Age in years		Total
	n (%)	mean SD	min–max	n (%)	mean SD	min–max	n (%)
Total	87 (51.8%)	23.6 ± 7.9	18–58	81 (48.2%)	26.6 ± 3.2	18–38	168 (100%)
General medical history							
General diseases	5 (3.0%)	33.6 ± 18.5	19–58	0 (0.0%)	0	0	5 (3.0%)
Impairment of wound healing	2 (1.2%)	22.0 ± 1.4	21–23	0 (0.0%)	0	0	2 (1.2%)
Infectious diseases	0 (0.0%)	–	–	0 (0.0%)	–	–	0 (0.0%)
Regular medication usage	7 (4.2%)	29.6 ± 16.6	19–58	2 (1.2%)	23.5 ± 2.1	22–25	9 (5.4%)
Oral homecare							
Daily tooth brushing							
1	21 (12.5%)	22.3 ± 4.1	18–34	25 (14.8%)	22.4 ± 2.6	18–27	46 (27.3%)
2	53 (31.5%)	23.8 ± 9.0	18–57	42 (25.0%)	22.4 ± 3.3	18–38	95 (56.5%)
≥ 3	13 (7.7%)	24.5 ± 8.2	18–49	14 (8.4%)	23.8 ± 3.8	20–32	27 (16.1%)
Private toothbrush	87 (51.8%)	23.6 ± 7.9	18–58	80 (47.6%)	22.6 ± 3.2	18–38	167 (99.4%)
Use of toothpaste	79 (47.0%)	23.8 ± 8.3	18–58	74 (44.0%)	22.5 ± 3.2	18–38	153 (91.0%)
Dental floss	2 (1.2%)	21 ± 0.0	21–21	4 (2.4%)	22.0 ± 0.8	21–23	6 (3.6%)
Toothpicks	1 (0.6%)	23 ± 0.0	23	5 (3.0%)	22.0 ± 1.7	21–25	6 (3.6%)
Toothache during daily tooth brushing	16 (9.5%)	26.7 ± 10.2	18–57	11 (6.6%)	21.1 ± 1.4	18–23	27 (16.1%)
Gingival bleeding while brushing	21 (12.5%)	23.5 ± 8.8	18–58	27 (16.1%)	22.7 ± 2.7	18–30	48 (28.6%)
Acute toothache	15 (8.9%)	25.7 ± 9.5	19–57	11 (6.6%)	22.2 ± 3.4	18–30	26 (15.5%)
Dental treatment							
Dental treatment within the two years	29 (17.3%)	24.5 ± 9.2	18–57	20 (11.9%)	23.5 ± 3.6	18–32	49 (29.2%)
Kind of treatment							
Fillings	4 (2.4%)	22.3 ± 2.2	20–25	1 (0.6%)	31.0 ± 0.0	31	5 (3.0%)
Extraction	23 (13.7%)	25.0 ± 9.9	18–57	14 (8.3%)	23.8 ± 3.9	18–32	37 (22.0%)
Dental check up	11 (6.6%)	25.9 ± 11.3	18–57	6 (3.6%)	23.3 ± 2.7	19–27	17 (10.2%)

### Dietary history

The results with regard to dietary history can be taken from Table 2. The majority (n = 140, 83%) of participants reported eating three meals per day. Eighty-seven (51.8%) participants did not eat any sweets. However, daily consumption of sugar-sweetened tea was reported by 153 (91.1%) of the participants. In addition, 93 (55.4%) participants reported daily consumption of soft drinks. Overall tobacco and alcohol consumption was very low according to the participants: only 5 (3.0%) participants ever consumed alcohol and only 2 (1.2%) participants ever consumed tobacco.

**Table 2:** Distribution of the responses of the nursing staff regarding their consumption of food and beverages

	Gender						
	Women	Age in years		Men	Age in years		Total
	n (%)	Mean SD	Min–max	n (%)	Mean SD	Min–max	n (%)
Total	87 (51.8%)	23.6 ± 7.9	18–58	81 (48.2%)	26.6 ± 3.2	18–38	168 (100%)
Dietary history							
Meals per day							
1	1 (0.6%)	23.0 ± 0.0	23	2 (1.2%)	24.0 ± 4.2	21–27	3 (1.8%)
2	15 (8.9%)	26.1 ± 10.9	19–57	10 (6.0%)	25.7 ± 5.6	18–38	25 (14.9%)
3	71 (42.2%)	23.0 ± 7.2	18–58	69 (41.1%)	22.1 ± 2.5	18–31	140 (83.3%)
Sweets per day							
0	43 (25.6%)	25.6 ± 10.4	18–58	44 (26.2%)	22.8 ± 3.5	18–38	87 (51.8%)
1	34 (20.2%)	21.6 ± 3.7	18–40	27 (16.1%)	22.8 ± 3.1	18–31	61 (36.3%)
2	10 (5.9%)	21.4 ± 1.3	19–23	5 (3.0%)	22.2 ± 0.84	21–23	15 (8.9%)
≥ 3	0 (0.0%)	–	–	5 (3.0%)	20.4 ± 1.5	18–22	5 (3.0%)
Softdrinks per day							
0	40 (23.8%)	23.7 ± 7.6	18–58	35 (20.8%)	22.4 ± 2.7	18–32	75 (44.6%)
1	46 (27.4%)	23.5 ± 7.9	18–57	40 (23.8%)	23.1 ± 3.7	18–38	86 (51.2%)
2	1 (0.6%)	19.0 ± 0.0	19	5 (3.0%)	21.0 ± 1.7	18–22	6 (3.6%)
> 2	0 (0.0%)	–	–	1 (0.6%)	20.0 ± 0.0	20	1 (0.6%)
Sugar-sweetened tea per day							
0	6 (3.6%)	28.8 ± 12.6	18–49	9 (5.3%)	22.2 ± 3.3	18–30	15 (8.9%)
1	69 (41.1%)	22.9 ± 6.7	18–58	65 (38.7%)	22.5 ± 3.2	18–38	134 (79.8%)
2	12 (7.1%)	24.3 ± 11.2	18–57	6 (3.6%)	24.7 ± 3.3	22–31	18 (10.7%)
> 2	0 (0.0%)	–	–	1 (0.6%)	21.0 ± 0.0	21	1 (0.6%)
Consumption of tobacco/alcohol							
Alcohol	2 (1.2%)	22.0 ± 1.4	21–23	3 (1.8%)	27.7 ± 9.3	20–38	5 (3.0%)
Tobacco	0 (0.0%)	–	–	2 (1.2%)	31.5 ± 9.2	25–38	2 (1.2%)

### Oral health data

**Fig. 1:**
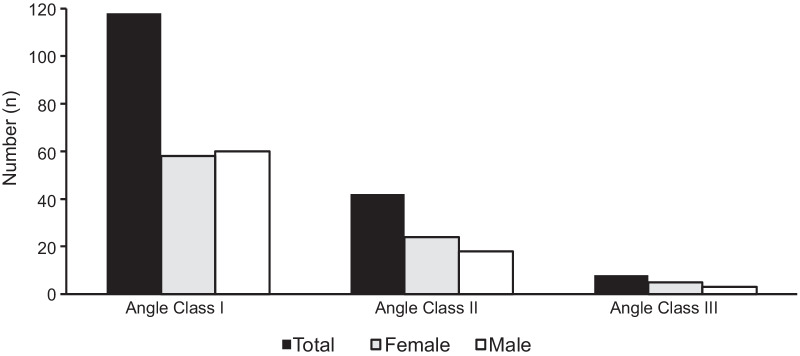
Absolute frequencies of angle classification of the participants subdivided into neutral, distal, and mesial occlusion

**Fig. 2:**
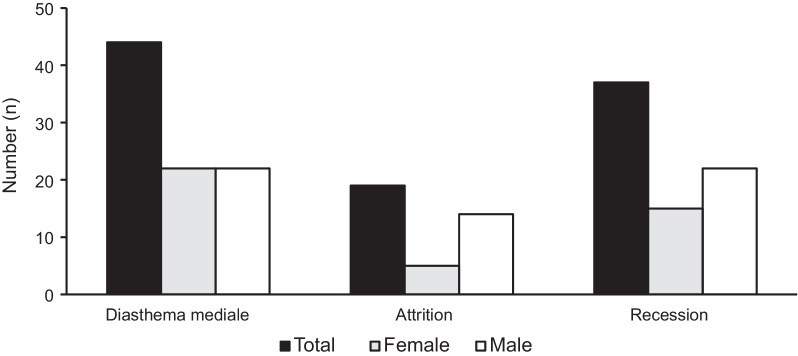
Absolute frequencies of intraoral characteristics of the participants subdivided into diastema, attrition, and recession

The results with regard to oral health data can be taken from Tables 3, 4, 5, 6, 7 and 8 and Figs. 1 and 2. The mean DMF/T-Index of the participants was 6.3 ± 4.5 and in 65 (38.7%) of the participants the caries incidence was very low (DMF/T-Index: 0–4.9) (Table 3). In the majority (n = 149, 88.7%) of the participants untreated decayed teeth (D) were identified (Table 4). The study participants’ age (*p* = 0.003), gender (*p* ≤ 0.001), angle class (*p* ≤ 0.001), frequency of teeth brushing (0.040), and the number of sweets per day (*p* = 0.013) had a significant influence on the DMF/T (Table 5). Nearly two-thirds (n = 106, 63.1%) of the participants showed an OHI-S value up to 1.2. The study participants’ age (*p* = 0.008), angle class (*p* = 0.011), and frequency of tooth brushing (*p* = 0.019) had a significant influence on OHI-S (Table 6). A neutral molar relationship (Angle-class I) was prevalent in the majority of the study participants (n = 118, 70.2%) (Fig. 1). Only a minority (in a maximum of 26.2% (n = 44) of the study participants showed specific intraoral characteristics like medial diastema, dental attrition and gingival recessions (Fig. 2). With the increase in age, the chance of recession and a diastema increased per year of life (Table 7). Two-thirds (n = 111, 66.1%) of the participants had no interrupted dental arch in either the maxilla or mandible (Table 8).

**Table 3:** Caries experience of the participants: Decayed, Missing, and Filled Teeth Index

	Gender								Total (n = 168)			
	Men (n = 81)				Women (n = 87)							
	Decayed teeth	missing teeth	filled teeth	DMF/T	Decayed teeth	missing teeth	filled teeth	DMF/T	Decayed teeth	missing teeth	filled teeth	DMF/T
Mean (SD)	4.31 (3.80)	1.04 (1.44)	0.04 (0.25)	5.38 (4,01)	4.53 (3.41)	2.51 (3.11)	0.13 (0.40)	7.15 (4.82)	4.42 (3.60)	1.80 (2.55)	0.08 (0.34)	6.30 (4.53)
Min–max	0–24	0–6	0–2	0–24	0–14	0–19	0–2	0–25	0–24	0–19	0–2	0–25
n	349	84	3	436	394	218	11	622	743	302	14	1058
Percentile values												
25	1.50	0.00	0.00	2.00	2.00	0.00	0.00	4.00	2.00	0.00	0.00	3.00
50 median	4.00	0.00	0.00	5.00	4.00	2.00	0.00	7.00	4.00	1.00	0.00	6.00
75	6.00	2.00	0.00	8.00	6.00	4.00	0.00	9.00	6.00	3.00	0.00	8.00

DMF/T, Decayed, Missing, and Filled Teeth

**Table 4:** Number of decayed teeth, missing teeth, and filled teeth distributed by gender

	Gender		Total (n)
	Women (n)	Men (n)	
Decayed (D)			
0	10	9	19
1	8	11	19
2	8	7	15
3	12	8	20
4	11	19	30
5	9	6	15
6	10	3	13
7	2	3	5
8	6	7	13
9	0	2	2
10	4	1	5
11	4	2	6
12	1	1	2
13	1	1	2
14	1	0	1
24	0	1	1
Missing (M)			
0	22	44	66
1	11	13	24
2	23	12	35
3	9	4	13
4	12	6	20
5	4	1	5
6	2	1	3
8	1	0	1
9	1	0	1
18	1	0	1
19	1	0	1
Filled (F)			
0	78	79	157
1	7	1	8
2	2	1	3
Total	87	81	168

**Table 5:** Factors associated with DMFT of the participants based on a linear regression analysis

R^2^ = 0.298	Regression coefficient B	95% confidence interval (CI) for B		SD	Beta	T	sig
		Min	Max				
(constant)	0.475	− 4.721	5.671	2.631		0.181	0.857
Gender	− 1.809	− 3.011	− 0.607	0.609	− 0.200	− 2.972	0.003
Age	0.250	0.150	0.350	0.051	0.339	4.928	0.000
Angle class	− 2.144	− 3.225	− 1.064	0.547	− 0.269	− 3.922	0.000
Dental care per day	0.938	0.043	1.832	0.453	0.141	2.071	0.040
Meals a day	0.100	− 1.334	1.533	0.726	0.010	0.137	0.891
Sweets per day	0.776	0.163	1.390	0.311	0.205	2.500	0.013
Sugar-sweetened tea per day	0.982	− 0.288	2.253	0.643	0.109	1.527	0.129
Soft drinks per day	0.310	− 0.898	1.519	0.612	0.040	0.508	0.612

**Table 6:** Factors associated with OHI-S of the participants based on a linear regression analysis

R^2^ = 0.153	Regression coefficient B	95% CI for B		SD	Beta	T	Sig
		Min	Max				
(Constant)	− 0.448	− 1.399	0.504	0.482		− 0.930	0.354
Gender	0.189	− 0.031	0.410	0.111	0.126	1.698	0.091
Age	0.025	0.006	0.043	0.009	0.202	2.672	0.008
Angle class	− 0.258	− 0.456	− 0.061	0.100	− 0.195	− 2.579	0.011
Dental care per day	0.197	0.033	0.361	0.083	0.178	2.379	0.019
Meals a day	0.260	− 0.003	0.522	0.133	0.149	1.953	0.053
Sweets per day	0.026	− 0.086	0.139	0.057	0.041	0.460	0.646
Sugar-sweetened tea per day	0.169	− 0.063	0.402	0.118	0.113	1.438	0.153
Soft drinks per day	0.040	− 0.182	0.261	0.112	0.031	0.354	0.724

**Table 7:** Factors associated with attritions, recessions and the occurrence of a diastema based on a binary logistic regression

	Regression coefficient B	SD	Wald	df	Sig	Exp(B)	95% CI for EXP(B)	
							Min	Max
Binary logistic regression for attritions								
Gender	1.096	0.625	3.073	1	0.080	2.992	0.879	10.185
Age	0.074	0.033	4.953	1	0.026	1.077	1.009	1.150
Constant	− 4.753	1.099	18.710	1	0.000	0.009		
Binary logistic regression for recessions								
Gender	0.703	0.393	3.206	1	0.073	2.019	0.936	4.358
Age	0.062	0.028	5.057	1	0.025	1.064	1.008	1.123
Constant	− 3.096	0.759	16.632	1	0.000	0.045		
Binary logistic regression for the occurrence of a diastema								
Gender	0.150	0.356	0.178	1	0.673	1.162	0.578	2.335
Age	0.040	0.026	2.234	1	0.135	1.040	0.988	1.096
Constant	− 2.034	0.691	8.660	1	0.003	0.131

**Table 8:** Incidence of Kennedy Class condition in absolute frequencies distributed in upper and lower jaw

	III	III/1	III/2	III/3	Total	DMF/T
Kennedy Class III						
Upper jaw	15	3	1	1	20	
Lower jaw	31	9	0	0	40	
Total	46	12	1	1	60	8.6

	II	II/1	II/2	II/3	Total	DMF/T
Kennedy Class II						
Upper jaw	0	1	0	0	1	
Lower jaw	2	2	0	0	4	
Total	2	3	0	0	5	11.6

	I	I/1	I/2	I/3	Total	DMF/T
Kennedy Class I						
Upper jaw	0	0	0	0	0	
Lower jaw	2	1	0	0	3	
Total	2	1	0	0	3	21

## Discussion

One hundred and sixty-eight nurses from ILH and nursing students of the IIHAS participated in this study. Both genders were equally represented. This was unexpected as in Tanzanian communities, nursing professionals are mostly women [5]; however, it strengthened the exploration of gender-specific dependencies.

The questionnaire was administered in Swahili and English. It was interview-guided to increase the response rate; that is, unanswered questions could be identified and answered immediately. Most participants (n = 162) reported that they did not suffer from general diseases. This could be due to the relatively low median age (22 years) of the participants in this study. Moreover, the entire anamnestic data collection could be causative for the low rate of general diseases. This may be especially true for the prevalence of infectious diseases. Therefore, the prevalence of HIV-positive participants is significantly lower than the average rate of infection in Tanzania (0% vs. 4.8%) [9]. Only 8.54% of the participants were taking medications. Question regarding the use of special types of medications were not asked.

The majority of the study participants (n = 167) had their own toothbrushes and brushed their teeth using toothpaste (n = 153) [10, 11]. In the present study, it was not investigated whether the toothpaste contained fluoride as an active ingredient. Most participants brushed their teeth twice daily (n = 95). Women brushed their teeth twice a day more often than men [12, 13]. In general, women showed increased health awareness compared to men, which can be considered under individual oral homecare [14]. The cleaning of interdental spaces was performed by only a few participants [5]. This scenario can be easily improved by implementing a structured individualized dental prophylaxes program in the educational curriculum of the IIHAS for complementing the oral health curriculum that was established in 1982 inprimary schools in Tanzania [10]. At the time of the investigation, an overwhelming majority of the study participants reported that they did not suffer from acute toothache. This finding suggests that toothache is a subjective symptom. In addition, the perception of pain depends on cultural and social factors [15]. Gum bleeding has been reported in every third participant (n = 48) [16]. More men suffered from gum bleeding than women. This may be partly because the oral homecare among men is not sufficient compared to that among women [14]. Another reason for increased gingival bleeding during home oral care could be incorrect toothbrushing technique. Using old or damaged toothbrushes could lead to an increase in gum problems even if the DMF/T-Index is low [17].

Nearly one-third of the participants reported that they had sought dental treatment within the two years prior to study participation [18]. The most commonly performed treatment was tooth extraction [19]. In the present study, nearly every tenth participant had participated in a dental check-up in the two years prior to the study inspection. This puts the study participants below the average achieved in other parts of Tanzania, where about 40% of respondents participated in an annual dental check-up [10]. This might be because preventive dental examinations, oral health education, and prophylactic dental treatments are not available in Tanzania or are hardly accepted by the population. The majority of the Tanzanian population uses dental treatment mostly for pain management [18, 20, 21]. Generally, the supply density of fully skilled dentists is very low in rural areas of Tanzania [6]. Therefore, it is extremely difficult for the Tanzanian population in rural areas to undergo regular dental check-ups [6].

Most study participants (83.3%) had three meals a day [17]. The consumption of sweets, sugar-sweetened tea, and soft drinks was at a higher level in the studied population [17]. A difference was observed in the habit of consuming highly sugar-sweetened foods. In East Africa, the consumption of sugar-sweetened foods is higher in urban than rural regions [22]. The increased consumption of soft drinks, sugar-sweetened tea, and sweets per day can lead to reduced oral health [23]. The consumption of alcohol and tobacco was very low in this study sample compared to average data in Tanzania [11, 21]. This could be because the consumption of alcohol is strictly prohibited on ILH premises and smoking is not allowed in public areas in Tanzania. Another reason for this result may be that the study participants were nurses and nursing students, so they were sensitive toward the health hazards of alcohol and tobacco consumption [24, 25].

The overall prevalence of caries in the population in this study was 88.7%. This value was significantly higher than other findings of dental caries in Tanzanian adults [5, 26]. However, the prevalence of dental caries, as expressed by the DMF/T index in this study, was lower than that in countries with higher income [27]. The overall mean DMF/T of the study participants was consistent with the reported findings in other Tanzanian adult populations [18]. Therefore, the DMF/T values of the study were based on the increased number of decayed teeth while the increased DMF/T-Indices of countries with an increased average age were due to the high number of filled teeth in the adult population [18]. Especially in rural areas of Tanzania, there is an increased prevalence of caries owing to the insufficient number of dental treatment facilities in rural areas compared to urban areas. This leads to a decreased frequency of dental check-ups to prevent initial carious lesions [28]. Therefore, the high DMF/T-Index can be explained by the increased values of decayed teeth and the small values of the missing and filled teeth of the participants. It must be mentioned that caries detection was performed visually in this study. Further dental tests or dental radiography could allow a more detailed specification of the caries level and the individual treatment demand of the participant. Higher caries risk is closely connected to the form and frequency of consumption of sugary foods, and the increase in frequency raises the risk of caries [29]. An increase in sugar consumption is reported to increase caries prevalence in low-income countries, and sugar consumption in Africa is increasing [30, 31]. The young adult age of the participants might have been causative for the caries prevalence in this study. Even in the rural areas of Tanzania, the supply density of modern sugar-containing foods has increased in the past decades, and young adults try to imitate the modern way of life of urban Western industrialized countries [32].

In the present study, the observed personal oral hygiene was good, but it was only half as good as the other reported rural areas [5]. Predominantly soft plaques were found in both women and men in this study. However, this led to high values of the Simplified Debris Index (DI/S), which were causal for the general OHI/S values. The older the study participants, the higher the OHI/S. The knowledge of personal oral hygiene is very limited in the wider population of Tanzania [5]. Nevertheless, it can be assumed that the study participants took part in the primary school oral health curriculum that was installed by the government of Tanzania in 1982 [10]. Therefore, it is expected that the study participants had basic knowledge about oral health. The good oral hygiene of the study participants was reflected by low DMF/T values in the international comparison [25]. The results of the personal oral hygiene should be detailed further in future studies using a coloring solution to describe the soft plaque areas of the participants.

The orthodontic treatment needs in this study were achievable. The most frequent orthodontic occlusion position was neutral occlusion, followed by distal occlusion, and rarely observed mesial occlusion [33, 34]. Here, the angle classification was used to describe the orthodontic treatment demands of the study participants. Furthermore, occlusion aberrations were not detected.

The incidence of diastema varies depending on the study region [35]. In the present study, a medial diastema of the upper jaw was recorded in one-quarter of the participants (n = 44). A medial diastema with a hereditary component without other oral malformations and further oral health restrictions do not lead to a dental or surgical treatment demand [35]. In 19 participants, dental attritions were identified, and the majority were men. The higher the age of the participants, the greater the number of dental attrition facets [36]. This finding is not surprising as dental attritions are caused by the consumption of acidic and sugary foods or untreated pairing functions. These are prevalent in older study populations [36]. Gingival recession was observed in one quarter of the participants with a high number among men. The prevalence of gingival recession correlated with the age of the participants. Therefore, this study yielded lower results owing to the young adult median age of the study participants [37]. Gingival recession might be associated with the presence of dental calculus and dental plaque, which may lead to an increasing prevalence of periodontal diseases [37]. The personal oral hygiene of the participants was found to be adequate in this study. This might have led to the generally low prevalence of gingival recession in this study in general and the increased prevalence of the same among men owing to their reduced personal oral hygiene.

The average tooth loss in the study participants was moderate. However, in nearly two-thirds (n = 111) of the participants, at least one tooth was missing, including wisdom teeth. These results are comparable and depend on the relatively young median age of the study participants [21]. Therefore, it is expected that the number of missing teeth increases with an increase in the median age of the population [1, 5, 13]. In the majority of the study participants, single-tooth gaps (Kennedy class III) were detected [1, 13, 38]. This predominant partially edentulous arch was detected more frequently in the lower jaw. Kennedy class IV was not observed in this study, which could be attributed to the young median age of the participants [38]. Tooth loss was more common in the women participants than men [13]. This is causative of the increased DMF/T values of women compared to men. The increased DMF/T-Index is based on untreated caries lesions which may result in an earlier personal tooth loss. Tooth extraction is the most common dental treatment in rural areas of Tanzania [21].

The study was limited to the nursing staff of the IIHAS and ILH in Ilembula, Tanzania. There was no randomization of the rural area or the hospital where the study was implemented. Therefore, no significance between the oral health situation of rural or urban regions could be considered. Moreover, it is challenging to transfer the impact of the results of the present study to the oral health situation of the surrounding population because the nursing staff might have different socio-economic backgrounds, levels of education, and availability of medical and dental treatment compared to the average population [31]. Nevertheless, the study was performed in February 2020 at the beginning of the COVID-19 pandemic. Therefore, the study could not be extended to the planned sample size owing to the travel restrictions.

## Conclusions

It can be concluded that most nursing staff of the ILH and IIHAS had good personal oral hygiene and low DMF/T values. The reported recall visits to dental treatment facilities were poor. However, the use of personal oral health items and practice of daily oral hygiene was better than expected. Against the background that the majority of Tanzanian adults mostly seek dental care for pain relief, it is important to sensitize nursing staff in rural areas regarding the prevention of oral diseases. Nurses in rural areas without adequate dental treatment facilities should screen and educate their patients regarding oral health-related diseases and how to prevent them. Therefore, advanced oral health knowledge should be inculcated into the structured education of nursing students. This study should be territorially extended to rural areas in Tanzania due to the lack of similar studies.

## Data Availability

The data used to support the findings of this study may be released upon an application to the Department of Prosthodontics, Martin-Luther-University Halle-Wittenberg, which can be contacted through Dr. Sebastian Hinz, Department of Prosthodontics, University Hospital Halle, Magdeburger Straße 16, 06112 Halle, Germany.

## Abbreviations

ILH: Ilembula Lutheran Hospital
IIHAS: Ilembula Institute of Health and Allied Sciences
DMF/T: Decayed, Missing, and Filled Teeth
OHI-S: Simplified Oral Hygiene Index
WHO: World Health Organization
OR: Odds ratio
CI: Confidence interval
DI/S: Simplified Debris Index
